# Supplementary material for: Obstructive sleep apnea increases risk of female infertility: A 14-year nationwide population-based study
Source: PLoS One. 2021 Dec 15;16(12):e0260842. doi: 10.1371/journal.pone.0260842 (PMC8673645; doi:10.1371/journal.pone.0260842)
Supplement: S1 Table — (DOCX) [file pone.0260842.s001.docx]

| **Table S1. Abbreviation and ICD-9-CM** | | |
| --- | --- | --- |
|  | **Abbreviation** | **ICD-9-CM** |
| **Study population:** Infertility |  | 628 |
| **Exposure:** Obstructive sleep apnea | OSAs | 327.23, 780.51, 780.53, 780.57 |
| **Exclusion criteria** |  |  |
| Radiation therapy | RT | V58.0 |
| Chemotherapy | CT | V58.1 |
| Genital organs related surgery |  | OP65-OP71 |
| Infertility causing factors |  |  |
| Malignant neoplasm of uterus |  |  |
| Malignant neoplasm of cervix uteri |  |  |
| Malignant neoplasm of placenta |  |  |
| Malignant neoplasm of body of uterus |  |  |
| Malignant neoplasm of ovary and other uterine adnexa |  |  |
| Uterine leiomyoma |  |  |
| Ovarian dysfunction |  |  |
| Infertility, male |  |  |
| Endometriosis |  |  |
| Menopausal and postmenopausal disorders |  |  |
| **Comorbidity** |  |  |
| Hypertension | HTN | 401-405 |
| Diabetes mellitus | DM | 250 |
| Hyperlipidemia |  | 272 |
| Chronic obstructive pulmonary disease | COPD | 490-496 |
| Chronic kidney disease | CKD | 585 |
| Ischemic heart disease | IHD | 410-414 |
| Congestive heart disease | CHD | 428-429 |
| Stroke |  | 438 |
| Cancer |  | 140-238 |
| Obesity |  | 278 |
| Hyperestrogenism |  | 256.0 |
| Polycystic ovaries |  | 256.4 |
| Irregular menstrual cycle |  | 626.4 |
| Endometriosis |  | 617 |
| Uterine leiomyoma |  | 218 |
| Cushing's syndrome |  | 255.0 |
| Thyrotoxicosis with or without goiter |  | 242 |
| Acquired hypothyroidism |  | 244 |
| Anxiety |  | 300.00 |
| Depression |  | 296.2-296.3, 300.4 |
