# Supplementary material for: Obstructive sleep apnea increases risk of female infertility: A 14-year nationwide population-based study
Source: PLoS One. 2021 Dec 15;16(12):e0260842. doi: 10.1371/journal.pone.0260842 (PMC8673645; doi:10.1371/journal.pone.0260842)
Supplement: S2 Table — (DOCX) [file pone.0260842.s002.docx]

| **Table S2. Correlation between variables listed in the table and age group** | | |
| --- | --- | --- |
| **Age group (yrs)** | **Correlation coefficient** | ***P*** |
| **Insured premium (NT$)** | 0.009 | 0.062 |
| **IHD** | 0.149 | <0.001 |
| **Cancer** | 0.121 | <0.001 |
| **Obesity** | 0.025 | 0.354 |
| **Hyperestrogenism** | No hyperestrogenism |  |
| **Polycystic ovaries** | No polycystic ovaries |  |
| **Irregular menstrual cycle** | 0.031 | 0.876 |
| **Endometriosis** | No endometriosis |  |
| **Uterine leiomyoma** | No uterine leiomyoma |  |
| **Anxiety** | -0.197 | 0.034 |
| **Depression** | -0.172 | 0.039 |
| **Season** | -0.069 | 0.157 |
| **Urbanization level** | -0.203 | <0.001 |
| **Level of care** | -0.148 | <0.001 |
| ***P:* Spearman correlation** | | |
