# Supplementary material for: Obstructive sleep apnea increases risk of female infertility: A 14-year nationwide population-based study
Source: PLoS One. 2021 Dec 15;16(12):e0260842. doi: 10.1371/journal.pone.0260842 (PMC8673645; doi:10.1371/journal.pone.0260842)
Supplement: Attachment — Submitted filename: Response to Reviewers.docx [file pone.0260842.s003.docx]

**Answer to Editor’s and Reviewer's comments**

Thank you for your positive comments on this manuscript. The responses to the raised questions are below.

The authors have serious and reasonable comments about this manuscript. The most compelling is the study design itself; this editor agrees that what the authors have shown is that amongst infertile women, OSA is more common, not that OSA is a potential cause of infertility. In addition, the editor would like to know how the diagnoses were searched for in the database. Were ICD codes used? If so, the authors need to indicate which codes were searched for (this could be done in a table).

**Response:** Thank you for your constructive critique. We totally agreed with you. OSA is more common, not that OSA is a potential cause of infertility. Due to it is a case control study, therefore, association of infertility and OSA was established in these 14 years follow up retrospective population base study. We used ICD-9-CM and added table S1 to indicate the codes.

| **Table S1. Abbreviation and ICD-9-CM** | | |
| --- | --- | --- |
|  | **Abbreviation** | **ICD-9-CM** |
| **Study population:** Infertility |  | 628 |
| **Exposure:** Obstructive sleep apnea | OSAs | 327.23, 780.51, 780.53, 780.57 |
| **Exclusion criteria** |  |  |
| Radiation therapy | RT | V58.0 |
| Chemotherapy | CT | V58.1 |
| Genital organs related surgery |  | OP65-OP71 |
| Infertility causing factors |  |  |
| Malignant neoplasm of uterus |  |  |
| Malignant neoplasm of cervix uteri |  |  |
| Malignant neoplasm of placenta |  |  |
| Malignant neoplasm of body of uterus |  |  |
| Malignant neoplasm of ovary and other uterine adnexa |  |  |
| Uterine leiomyoma |  |  |
| Ovarian dysfunction |  |  |
| Infertility, male |  |  |
| Endometriosis |  |  |
| Menopausal and postmenopausal disorders |  |  |
| **Comorbidity** |  |  |
| Hypertension | HTN | 401-405 |
| Diabetes mellitus | DM | 250 |
| Hyperlipidemia |  | 272 |
| Chronic obstructive pulmonary disease | COPD | 490-496 |
| Chronic kidney disease | CKD | 585 |
| Ischemic heart disease | IHD | 410-414 |
| Congestive heart disease | CHD | 428-429 |
| Stroke |  | 438 |
| Cancer |  | 140-238 |
| Obesity |  | 278 |
| Hyperestrogenism |  | 256.0 |
| Polycystic ovaries |  | 256.4 |
| Irregular menstrual cycle |  | 626.4 |
| Endometriosis |  | 617 |
| Uterine leiomyoma |  | 218 |
| Cushing's syndrome |  | 255.0 |
| Thyrotoxicosis with or without goiter |  | 242 |
| Acquired hypothyroidism |  | 244 |
| Anxiety |  | 300.00 |
| Depression |  | 296.2-296.3, 300.4 |

Reviewer #1:

Major points:

1. This study draws subjects from a large database, however, the number of with infertility and OSA was only 33 with only 30 subjects with OSA and no infertility in the control group.

**Response:** Thank you for your thorough review and salient observations. The limited number of subjects in our study is because we have excluded women who received radiation therapy, chemotherapy, genital organ surgery, and aged <20, >45 years old. We identified women who became infertile and created a 2:1 matched control group with women who were not infertile, and got 4,800 of fertility control and 2,400 of infertility case. After that, we traced back to their OSA exposure, with 33 subjects in the case group and 30 subjects in the control group.

2. It is not clear what prior exposure to OSA means. Is this the presence of disease at the start of the study, the development of OSA during the time of the study, or both? Similarly, it’s not clear what the “years of OSA exposure” is referring to. I’m not sure what Table 4 is trying to tell me.

**Response:** Thank you for your thorough review and salient observations. Our study is a case control study which firstly identified infertile women, then traced back to their history of OSA. What table 4 (now table 3) is trying to say is that the more years of having OSA, the higher the risk of having infertility.

3. The groups were then stratified into five-year age groups, making the numbers even smaller and the conclusions less robust.

**Response:** Thank you for your thorough review and salient observations.

Although the number is lesser in stratified groups, we have the largest participants (N=989,753) from NHIRD database, which has not been previously reported. And we have adjusted the odd ratio by multivariable conditional logistic regression, which we think that the p value of each stratified variables was representative. The reason why we stratified into five-year age groups is because we wanted to dig deeper into the age-related effect on OSA and infertility and provide a greater precision, rather than the entire age group.

4. The definition of infertility includes a time period of at least one year. How would the authors know which season has the highest risk for infertility?

**Response:** Thank you for your thorough review and salient observations.

This is a 14-year follow-up case control study, first identified the infertility then retrospectively found out previous exposure of OSA, therefore, infertility was definitely diagnosed after a time period of at least one year. We also divided 365 days into four seasons for the first time of infertility diagnosis.

5. The limitations section is very brief and should address the issue of the small sample sizes of the OSA/infertility and OSA/non-infertility groups.

**Response:** Thank you for your thorough review and salient observations. We have revised these mistakes based on your recommendation in limitations section.

Third, the study has the small sample size after retrospectively extracted previous OSA exposure from participants with infertility and no infertility, however, the study has the largest database (N=989,753), which has not been previously reported.

6. The authors conclude that detection and diagnosis of OSA may help to increase fertility, but this is speculative. There is no clear data to support this.

**Response:** Thank you for your thorough review and kind suggestions. Indeed, there is no clear data to date about the association between OSA and female infertility. Therefore, we have performed this retrospective study to proof this hypothesis. In our study, we have found increased risk of female infertility in OSA patients. However, as this is a case control study, we could only determine the association between infertility and OSA. Therefore, we revised these mistakes based on your recommendation in Conclusion part.

In conclusion, this study showed OSA is more commonly seen in infertile women,

especially those with concomitant comorbidities.

Minor points:

1. Many acronyms are not defined when first used: NHIRD, NASD, PCOS, COPD.

**Response:** Thank you for your thorough review and salient observations. We have revised these mistakes based on your recommendation by adding abbreviation list.

Abbreviation list:

NHIRD, National Health Insurance Research Database

NASD, Non-apnea sleep disorders

PCOS, Polycystic ovary syndrome

COPD, Chronic obstructive pulmonary disease

OSA, Obstructive sleep apnea

ICD-9-CM, International Classification of Diseases, Ninth Revision, Clinical Modification

ACOG, American College of Obstetricians and Gynecologists

2. The last sentence of the “Study Population” section is incomplete.

**Response:** Thank you for your thorough review and salient observations. We have revised these mistakes based on your recommendation

Moreover, common comorbidities as well as endocrine and gynecological diseases, such as obesity, Cushing syndrome, thyroid disease, PCOS, and endometriosis, were used in the analysis to evaluate the cause of infertility and their effects on OSA.

3. Under “Results,” a sentence reads “Women aged 26-30 years… had the highest percentage.” The highest percentage of what?

**Response:** Thank you for your thorough review and salient observations. We have revised these mistakes based on your recommendation.

Women aged 26-30 years and 31-35 years were more than other aged groups in our study population (28.0% and 32.29% respectively).

4. Reference 21 in the manuscript does not appear to refer to reference 21 in the References section.

**Response:** Thank you for your thorough review and salient observations. We have revised these mistakes based on your recommendation.

We have deleted reference 21.

5. in the discussion section, the word choice of “elderly” is not appropriate, since elderly women are clearly post-menopausal and not candidates for infertility.

**Response:** Thank you for your thorough review and salient observations. We have revised these mistakes based on your recommendation.

In the general population, older women are more likely to be infertile than younger women.

6. There is a sentence in the discussion that reads, The increased of cortisol…” A word appears to be missing.

**Response:** Thank you for your thorough review and salient observations. We have revised these mistakes based on your recommendation.

The increased of cortisol levels downregulated the hypothalamic-pituitary-adrenal axis and involved in the inhibition of GnRH at the pituitary level, which altered sex hormone profile and thus leading to infertility.

7. Under the Results section, the Tables are mislabeled.

**Response:** Thank you for your thorough review and salient observations. We have revised these mistakes based on your recommendation (page 10-11).

Reviewer #2:

MAJOR WEAKNESSES

General:

1. There is confusion as to what the authors are trying to prove. They conclude that women with OSA are more likely to be infertile but what they show is that women who are infertile have greater odds of having OSA than women who are fertile. This study is not the same design as the prior work, where they looked at women with NASD and then did a prospective study to see the risk of infertility.

**Response:** Thank you for your thorough review and salient observations.

Our previous study was a prospective study but present study is a case-control study.

2. They do not mention how they define infertility nor if they excluded male infertility.

**Response:** Thank you for your thorough review and salient observations. We have revised these mistakes based on your recommendation in introduction section.

(1) According to the American College of Obstetricians and Gynecologists (ACOG), the definition of infertility is the failure to conceive after 1 year or more of regular unprotected sexual intercourse, <https://www.acog.org/womens-health/faqs/treating-infertility>.

(2) We extracted data from female infertility which was definitely diagnosed in these 14 years follow up case control study after male infertility has been excluded.

3. It is surprising that a cohort of 2400 women with infertility has no cases of endometriosis (with an estimated prevalence of 25-50% among infertile women) or PCOS (present in about 40% of women with infertility) making generalizability of any conclusions questionable.

**Response:** Thank you for your thorough review and salient observations.

We totally agreed with you. The estimated prevalence of endometriosis and PCOS were 25-50% and 40% among infertile women.

Prevalence is based on both incidence and duration of illness. High prevalence of a disease within a population might reflect high incidence or prolonged survival without cure or both. Thus, prevalence rate is always higher than incidence rate [6]. Endometriosis and PCOS were treatable disease [1-5], therefore, it may also be the reason of no cases of endometriosis and PCOS in our cohort of 2400 women with infertility.

However, we agreed to your precise recommendation as the limitation of our study and have revised these mistakes based on your recommendation in Limitation part. Fourth, there has no incidence of endometriosis or PCOS among infertile women in case group.

References:

1. Küpker W, Felberbaum RE, Krapp M, Schill T, Malik E, Diedrich K. Use of GnRH antagonists in the treatment of endometriosis. Reprod Biomed Online. 2002 Jul-Aug;5(1):12-6. doi: 10.1016/s1472-6483(10)61590-8. PMID: 12470539.

2. Rabinovici J, Stewart EA. New interventional techniques for adenomyosis. Best Pract Res Clin Obstet Gynaecol. 2006 Aug;20(4):617-36. doi: 10.1016/j.bpobgyn.2006.02.002. Epub 2006 Aug 24. PMID: 16934530.

3. Hirsch M, Dhillon-Smith R, Cutner AS, Yap M, Creighton SM. The Prevalence of Endometriosis in Adolescents with Pelvic Pain: A Systematic Review. J Pediatr Adolesc Gynecol. 2020 Dec;33(6):623-630. doi: 10.1016/j.jpag.2020.07.011. Epub 2020 Jul 29. PMID: 32736134.

4. Khadilkar S. S. (2019). Can Polycystic Ovarian Syndrome be cured? Unfolding the Concept of Secondary Polycystic Ovarian Syndrome!. Journal of obstetrics and gynaecology of India, 69(4), 297–302. <https://doi.org/10.1007/s13224-019-01253-z>

5. Arain F, Arif N, Halepota H. Frequency and outcome of treatment in polycystic ovaries related infertility. Pak J Med Sci. 2015;31(3):694-9. doi: 10.12669/pjms.313.8003. PMID: 26150870; PMCID: PMC4485297.

6. https://www.cdc.gov/csels/dsepd/ss1978/lesson3/section2.html

4. There is no discussion of how they determined the duration of the diagnosis of OSA relative to the timing of the diagnosis of infertility.

**Response:** Thank you for your thorough review and salient observations. We have added table 4 to determine the duration of the diagnosis of OSA relative to the timing of the diagnosis of infertility.

| **Table 4. Factors of infertility stratified by timing of the diagnosis of infertility by using conditional logistic regression** | | | | |
| --- | --- | --- | --- | --- |
|  | **With OSAs *vs*. without OSAs** *(Reference)* | | | |
| **The last OSAs exposure to the first infertility diagnosis** | **Adjusted OR** | **95%CI** | **95%CI** | ***P*** |
| **Overall** | 2.101 | 1.118 | 3.950 | <0.001 |
| < 1 year | 3.861 | 2.750 | 4.895 | <0.001 |
| ≧1 year, < 5 years | 2.450 | 1.598 | 3.376 | <0.001 |
| ≧5 years | 1.786 | 1.049 | 2.385 | 0.001 |
| **Adjusted OR = Adjusted odds ratio: Adjusted for the variables listed in Table 2.; CI = confidence interval** | | | | |

5. The abstract suggests a different protocol, which would have been to look at women with OSA and see how many are infertile while the actual protocol was to determine odds ratio of having OSA in a cohort of subjects who are infertile.

**Response:** Thank you for your thorough review and salient observations. We have revised these mistakes based on your recommendation in the abstract.

To determine the risk of having obstructive sleep apnea in a cohort of female patients who are infertile.

Introduction:

6. There is a discussion about effect of physiological consequences of OSA on sperm production in mice. This does not seem relevant to a paper on female infertility. Consider discussing more about female-relevant hormones, such as you do in the discussion.

**Response:** Thank you for your thorough review and salient observations. We have revised these mistakes based on your recommendation. We have revised the sentence that related to sperm production in mice to “Scientists have demonstrated young to middle-aged mice which experienced chronic intermittent hypoxia, have reduced the reproductive functions via the decreased of nitric oxide production” and added 4 new references to discuss more about female-relevant hormones in discussion part.

In addition, sex hormone also participated in the distribution of adipose tissue and muscle function in upper respiratory tract [1]. Scientist have demonstrated that high progesterone level in pregnancy status had the protective role in developing OSA, even in obese pregnant women [2, 3]. Greater activity of dilator muscle in upper respiratory tract was also seen in progesterone-dominant luteal phase [4].

References:

1. Fotis Kapsimalis, MD, Meir H. Kryger, MD, FRCPC, Gender and Obstructive Sleep Apnea Syndrome, Part 2: Mechanisms, Sleep, Volume 25, Issue 5, August 2002, Pages 497–504, https://doi.org/10.1093/sleep/25.5.497

2. Brownell LG, West P, Kryger MH. Breathing during sleep in normal pregnant women. Am Rev Respir Dis. 1986;133(1):38-41.

3. Maasilta P, Bachour A, Teramo K, Polo O, Laitinen LA. Sleeprelated disordered breathing during pregnancy in obese women. Chest.

2001;120(5):1448-54.

4. Popovic RM, White DP. Upper airway muscle activity in normal women: influence of hormonal status. J Appl Physiol 1998;84(3):1055-1062.

Study Population:

7. Did you exclude women who were infertile because of male infertility issues?

Response: Thank you for your thorough review and salient observations. We extracted data from female infertility which was definitely diagnosed in these 14 years follow up case control study after male infertility has been excluded.

8. How did you determine the prior exposure of OSA?

**Response:** Thank you for your thorough review and salient observations. We determined the prior exposure of OSA by the first infertility diagnosis. We have added table 4 to determine the duration of the diagnosis of OSA relative to the timing of the diagnosis of infertility.

| **Table 4. Factors of infertility stratified by timing of the diagnosis of infertility by using conditional logistic regression** | | | | |
| --- | --- | --- | --- | --- |
|  | **With OSAs *vs*. without OSAs** *(Reference)* | | | |
| **The last OSAs exposure to the first infertility diagnosis** | **Adjusted OR** | **95%CI** | **95%CI** | ***P*** |
| **Overall** | 2.101 | 1.118 | 3.950 | <0.001 |
| < 1 year | 3.861 | 2.750 | 4.895 | <0.001 |
| ≧1 year, < 5 years | 2.450 | 1.598 | 3.376 | <0.001 |
| ≧5 years | 1.786 | 1.049 | 2.385 | 0.001 |
| **Adjusted OR = Adjusted odds ratio: Adjusted for the variables listed in Table 2.; CI = confidence interval** | | | | |

9. Last sentence is incomplete.

**Response:** Thank you for your thorough review and salient observations. We have revised these mistakes based on your recommendation. We have deleted the unnecessary words.

Statistical Analysis

10. There is mention of a Table 5 that is not included in the submission.

**Response:** Thank you for your thorough review and salient observations. We have revised these mistakes based on your recommendation. We have added table 5 (now table S2) in the end of manuscripts.

| **Table S2. Correlation between variables listed in the table and age group** | | |
| --- | --- | --- |
| **Age group (yrs)** | **Correlation coefficient** | ***P*** |
| **Insured premium (NT$)** | 0.009 | 0.062 |
| **IHD** | 0.149 | <0.001 |
| **Cancer** | 0.121 | <0.001 |
| **Obesity** | 0.025 | 0.354 |
| **Hyperestrogenism** | No hyperestrogenism |  |
| **Polycystic ovaries** | No polycystic ovaries |  |
| **Irregular menstrual cycle** | 0.031 | 0.876 |
| **Endometriosis** | No endometriosis |  |
| **Uterine leiomyoma** | No uterine leiomyoma |  |
| **Anxiety** | -0.197 | 0.034 |
| **Depression** | -0.172 | 0.039 |
| **Season** | -0.069 | 0.157 |
| **Urbanization level** | -0.203 | <0.001 |
| **Level of care** | -0.148 | <0.001 |
| ***P:* Spearman correlation** | | |

Results:

11. Table 3 is confusing—consider deleting these data. Table 2 is sufficient

**Response:** Thank you for your thorough review and salient observations. We have deleted Table 3 based on your recommendation.

Discussion:

12. You did not demonstrate that women with OSA had twice the risk of infertility but, rather, that infertile women had an odds ratio of 2.1 of having OSA compared with women who were fertile.

**Response:** Thank you for your thorough review and salient observations. We have revised these mistakes based on your recommendation by substitute the sentence to “In this nationwide, population-based, case-control study of over 14 years, we have found that infertile women had an odds ratio of 2.1 of having OSA compared with women who were fertile by creating a 2:1 matched control group with women who were not infertile”, instead of “women with OSA had twice the risk of infertility” (page 12).

13. The fifth paragraph is confusing and the conclusion does not follow. The sex hormones do not mask the severity—they influence the severity.

**Response:** Thank you for your thorough review and salient observations. We totally agreed with you. We have revised “Sex hormones do not mask the severity” to

sex hormones may influence the severity of OSA (page 15).

MINOR WEAKNESSES

Suggested Content Edits:

Study Population:

1. “The diagnosis of OSA is made BY polysomnography . . . .”

**Response:** Thank you for your thorough review and salient observations. We have revised these mistakes based on your recommendation (page 16).

Statistical Analysis:

2. “Correlation[s] analysis WAS used”

**Response:** Thank you for your thorough review and salient observations. We have revised these mistakes based on your recommendation (page 10).

3. “Women aged 26-30 years and 31-35 years had the highest percentage”. I believe you mean “constituted the highest percentage”. If this is not what you mean, then please state what they had the highest percentage of.

**Response:** Thank you for your thorough review and salient observations. We have revised these mistakes based on your recommendation.

Women aged 26-30 years and 31-35 years were more than other aged groups in our study population (28.0% and 32.29% respectively).

Discussion,

4. 1st paragraph, last sentence: “This article is the second study that our groups have shown that women . . . infertility”. Consider substituting instead “This article is the second study in which our group has shown that women with sleep disorders . . . infertility.”

**Response:** Thank you for your thorough review and salient observations. We have revised these mistakes based on your recommendation (page 12).

5. 3rd paragraph: “. . . elderly women are more likely to become infertile”—I believe you want to say “. . . OLDER women are more likely to BE infertile”.

**Response:** Thank you for your thorough review and salient observations. We have revised these mistakes based on your recommendation (page 13).

Endnotes:

6. Endnotes 31 and 32 do not relate to the sentence they are in.

**Response:** Thank you for your thorough review and salient observations. We have revised these mistakes based on your recommendation.

We revised the reference to “Potter GD, Skene DJ, Arendt J, Cade JE, Grant PJ, Hardie LJ. Circadian rhythm and sleep disruption: causes, metabolic consequences, and countermeasures. Endocrine reviews 2016; 37(6): 584-608.”

Last, we are deeply honored by the time and effort you spent in reviewing this manuscript. In reviewing and revising our text, we are motivated to read more and thus learn more from your criticisms.
