# Supplementary material for: Obstructive sleep apnea increases risk of female infertility: A 14-year nationwide population-based study
Source: PLoS One. 2021 Dec 15;16(12):e0260842. doi: 10.1371/journal.pone.0260842 (PMC8673645; doi:10.1371/journal.pone.0260842)
Supplement: Attachment — Submitted filename: 2021-09-21 Response to Reviewers.docx [file pone.0260842.s004.docx]

**Answer to Editor’s and Reviewer's comments**

Thank you for your positive comments on this manuscript. The responses to the raised questions are below.

Reviewer #2: Thank you for your work addressing both my concerns and those of the other reviewer. This revision makes it more clear that you are attempting to do two different analyses, which still needs to be clearly enunciated (see comments below). In addition, I am still concerned and confused about the absence of cases of endometriosis and the presence of just one case of PCOS (which happened to be in the fertile cohort). Please address these issues directly, particularly as I found an analysis of the LHID covering a similar period of time that was published in 2019 (https://www.liebertpub.com/doi/full/10.1089/jwh.2018.7648) looking at the relationship of PCOS and periodontal disease that identified 744 patients with PCOS and it is surprising that not a single one of these cases also had a diagnosis of infertility and did not have one of your exclusionary criteria. Finally, I have some issues with your description about the timing of OSA diagnosis and onset of infertility and I believe that the discussion about seasonality is less relevant.

**Response:** Thank you for your constructive critique. We have revised one by one according to your recommendations below.

MAJOR WEAKNESSES

ABSTRACT

Objectives: There are actually two objectives: to determine the risk of having OSA in a cohort of female subjects who are infertile and the odds of being infertile in women with OSA.

Results: Highlight the disparity between the prevalence of OSA in the infertile versus fertile group: “Of those women with infertility, 1.38% had a history of OSA compared with 0.63% of fertile controls (p=0.002).

Conclusion: You did not show that OSA is underdiagnosed nor did you fully show that if untreated, it increases the likelihood of infertility. Please be more precise: “Our study showed that OSA is more commonly seen in infertile women and increases the odds that a woman will be infertile. In addition, the likelihood of being diagnosed with infertility increases with time from the diagnosis of OSA [although please see my discussion about this particularly factor below]. More studies need to be done on the whether or not diagnosing and treating OSA can decrease the rate of infertility.”

**Response:** Thank you for your thorough review and salient observations. We have revised them according to your recommendation.

**Objectives:** To determine the risk of having OSA in a cohort of female subjects who are infertile and the odds of being infertile in women with OSA.

**Patients and methods:** A nationwide, case-control study of female patients 20 years or older diagnosed with female infertility living in Taiwan, from January 1, 2000, through December 31, 2013 (N = 4,078). We identified women who were infertile and created a 2:1 matched control group with women who were not infertile. We used multivariable logistic regression analysis to further estimate the effects of OSA on female infertility.

**Results:** In this 14- year retrospective study, we included 4,078 patients having an initial diagnosis of female infertility. Of those women with infertility, 1.38% had a history of OSA compared with 0.63% of fertile controls (p=0.002). The mean ages in the study groups were 32.19 ± 6.20 years, whereas the mean ages in the control groups were 32.24 ± 6.37years. Women with OSA had 2.101- times the risk of female infertility compared to women without OSA (p<0.001).

**Conclusion:** Our study showed that OSA is more commonly seen in infertile women and increases the odds that a woman will be infertile. More studies need to be done on the whether or not diagnosing and treating OSA can decrease the rate of infertility.

INTRODUCTION

Third Paragraph: Please delete the whole section about mice as it is irrelevant and relates to male mice.

**Response:** Thank you for your thorough review and salient observations. We have deleted the whole section about mice as it is irrelevant and relates to male mice.

Infertility can be associated with multiple factors, such as inflammation, obesity, intermittent hypoxia and sympathetic activation^8,9^. In addition, continuous positive airway pressure treatment showed the increased rate of successful intercourse attempts and increased of the score in International Index of Erectile Function ^10,11^. In our previous study, we have found an increased risk of infertility in female patients with non-apnea sleep disorder ^12^. However, little is known about whether OSA is associated with a risk of female infertility.

References:

8.	Torres M, Laguna-Barraza R, Dalmases M, et al. Male fertility is reduced by chronic intermittent hypoxia mimicking sleep apnea in mice. Sleep 2014; 37(11): 1757-65.

9.	Soukhova-O'Hare GK, Shah ZA, Lei Z, Nozdrachev AD, Rao CV, Gozal D. Erectile dysfunction in a murine model of sleep apnea. American journal of respiratory and critical care medicine 2008; 178(6): 644-50.

10.	Li X, Dong Z, Wan Y, Wang Z. Sildenafil versus continuous positive airway pressure for erectile dysfunction in men with obstructive sleep apnea: a meta-analysis. The Aging Male 2010; 13(2): 82-6.

11.	Perimenis P, Karkoulias K, Konstantinopoulos A, et al. Sildenafil versus continuous positive airway pressure for erectile dysfunction in men with obstructive sleep apnea: a comparative study of their efficacy and safety and the patient's satisfaction with treatment. Asian journal of andrology 2007; 9(2): 259-64.

12.	Wang ID, Liu YL, Peng CK, et al. Non-apnea sleep disorder increases the risk of subsequent female infertility-a nationwide population-based cohort study. Sleep 2018; 41(1): zsx186.

RESULTS

Second Paragraph: You should have the increased likelihood of subjects with OSA to be infertile at the end of this paragraph where you discuss the other results from Table 2 rather than coming after the information about stratification by possible years of exposure to OSA that starts paragraph 3.

**Response:** Thank you for your thorough review and salient observations. We have revised according to your recommendation.

Multivariable logistic regression analysis was used in this retrospective study. We listed factors for female infertility in Table 2. There were no significant differences in the risk of female infertility between patients with and without gynecological disorders, endocrine disorders, or concomitant comorbidities, including hypertension, diabetes mellitus, hyperlipidemia, COPD, chronic kidney disease, coronary heart disease, stroke, obesity, anxiety, and depression (Table 2). The increased likelihood of subjects with OSA to be infertile was also showed in Table 2 (adjusted odds ratio, 2.101; p<0.001).

Third Paragraph: I would eliminate the discussion about years of prior OSA exposure since you do not know which patients, if any, were treated nor do you know severity, rendering this a statistically significant result that may or may not have clinical relevance. It is enough to establish an association between having a diagnosis of OSA and one of infertility unless you had more information. Also, Tables 3 and 4 are confusing—it is not clear what you mean about first and last exposure to OSA prior to diagnosis of infertility.

**Response:** Thank you for your thorough review and salient observations. We have deleted them according to your recommendation.

DISCUSSION

First Paragraph: You not only found that women with OSA had an odds ratio of 2.1 of being infertile but also that infertile women were more likely to have OSA.

**Response:** Thank you for your thorough review and salient observations. We have revised according to your recommendation. Our study is the largest retrospective study to date demonstrating the association of female infertility with OSA. In this nationwide, population-based, case-control study of over 14 years, we have found that infertile women had an odds ratio of 2.1 of having OSA compared with women who were fertile **but also infertile women were more likely to have OSA.** This article is the second study in which our groups has shown that women with sleep disorders could link to female infertility. In our previous article, we have found that women with non-apnea sleep disorder had a 3.718-fold risk of female infertility compared with the control cohort^12^.

Reference:

12.	Wang ID, Liu YL, Peng CK, et al. Non-apnea sleep disorder increases the risk of subsequent female infertility-a nationwide population-based cohort study. Sleep 2018; 41(1): zsx186.

Third Paragraph: I do not think it adds much and would consider deleting it.

**Response:** Thank you for your thorough review and salient observations. We have deleted third paragraph according to your recommendation.

Fourth Paragraph:

1. Is it clear that OSA increases the risk of PCOS or, rather, that patients with PCOS are at greater risk of having OSA, not just because of obesity but also because of hyperadrenergism? Also, I am not aware of OSA increasing the risk of endometriosis. Reference 29 talks about progesterone—I do not think it addresses what you wrote.

**Response:** Thank you for your thorough review and salient observations. PCOS was defined as 1) oligoovulation, 2) clinical hyperandrogenism (i.e. hirsutism) and/or hyperandrogenemia, and 3) exclusion of other related disorders, such as hyperprolactinemia, thyroid abnormalities, and non-classic adrenal hyperplasia^1^. Therefore, PCOS are at greater risk of having OSA, not just because of obesity but also because of hyperadrenergism. Also, due to OSA did not increase the risk of endometriosis, we have deleted the word “endometriosis” according to your suggestion.

References:

1. Knochenhauer, E. S., Key, T. J., Kahsar-Miller, M., Waggoner, W., Boots, L. R., & Azziz, R. (1998). Prevalence of the polycystic ovary syndrome in unselected black and white women of the southeastern United States: a prospective study. The Journal of clinical endocrinology and metabolism, 83(9), 3078–3082. <https://doi.org/10.1210/jcem.83.9.5090>

2. Reference 32 describes the presentation of sleep-disordered breathing in women and does not discuss the HPA axis and infertility. Either delete this sentence or put in the correct reference. Perhaps you mean to be referencing reference 32 from the initial draft and the article by Ferin?

**Response:**

Thank you for your thorough review and salient observations. We have corrected the reference to Ferin M. Stress and the Reproductive Cycle. The Journal of Clinical Endocrinology & Metabolism 1999; 84(6): 1768-74.

3. The last 2 sentences seem relevant as this is not a study of salivary cortisol.

**Response:** Thank you for your thorough review and salient observations. We have revised the reference according to your suggestion.

Patients with OSA are at risk of metabolic disorders, including an irregular menstrual cycle, obesity and PCOS^29^. The menstrual cycle is modulated by the hypothalamus–pituitary–gonadal axis. The hypothalamus releases gonadotropin-releasing hormone, the pituitary gland produces follicle-stimulating hormone and luteinizing hormone, and the ovary produces estrogen and testosterone. Kloss et al. hypothesized that sleep disorders may activate the hypothalamus–pituitary–gonadal axis and alter sex hormones in the follicular, ovulation, luteal, and menstruation phases, ultimately resulting in inferility^30^. In addition, the interruption of breathing during sleep as a result of OSA can cause circadian dysrhythmia due to the increased levels of melatonin and cortisol^31^. The increased of cortisol levels downregulated the hypothalamic-pituitary-adrenal axis and involved in the inhibition of GnRH at the pituitary level, which altered sex hormone profile and thus leading to infertility^32^. Moreover, women with higher salivary cortisol levels have lower levels of estradiol during the follicular and luteal phases, accompanied by an increased concentration of cortisol and estrogen receptors in the mood regulation region of the brain**^33^**. Therefore, salivary cortisol levels are inversely proportional to estradiol levels, which may lead to missed or irregular periods and make getting pregnant difficult.

References:

29.	Nitsche K, Ehrmann DA. Obstructive sleep apnea and metabolic dysfunction in polycystic ovary syndrome. Best Pract Res Clin Endocrinol Metab 2010; 24(5): 717-30.

30.	Kloss JD, Perlis ML, Zamzow JA, Culnan EJ, Gracia CR. Sleep, sleep disturbance, and fertility in women. Sleep Med Rev 2015; 22: 78-87.

31.	Potter GDM, Skene DJ, Arendt J, Cade JE, Grant PJ, Hardie LJ. Circadian Rhythm and Sleep Disruption: Causes, Metabolic Consequences, and Countermeasures. Endocr Rev 2016; 37(6): 584-608.

32.	Ferin M. Stress and the Reproductive Cycle. The Journal of Clinical Endocrinology & Metabolism 1999; 84(6): 1768-74.

**33.	Hamidovic A, Karapetyan K, Serdarevic F, Choi SH, Eisenlohr-Moul T, Pinna G. Higher Circulating Cortisol in the Follicular vs. Luteal Phase of the Menstrual Cycle: A Meta-Analysis. Front Endocrinol (Lausanne) 2020; 11: 311-.**

Fifth Paragraph:

Consider deleting this. It is not relevant to your argument and has some confusing concepts. For example, the lower prevalence of OSA In women is not because they have different presenting symptoms, it is because of other reasons you mention.

**Response:** Thank you for your thorough review and salient observations. We have deleted it according to your recommendation.

Sixth Paragraph:

I have difficulty with the concept of determining an association of the season of onset of infertility with the date of diagnosis so I would eliminate this entire finding/discussion.

**Response:** Thank you for your thorough review and salient observations. We have deleted it according to your recommendation.

Seventh Paragraph/Limitations:

1. You need to mention that when assessing the duration of OSA, it is approximated by date of diagnosis code and that you do not know whether or not it is treated subsequent to that. I would delete the analysis of this factor because of the limited knowledge but if you keep it in, you need to mention this as a limitation.

**Response:** Thank you for your thorough review and salient observations. We have deleted the analysis of this factor and added it as limitation, according to your recommendation.

This study had some limitations. First, our database could not provide detailed information about laboratory data or about the psychological impact of infertility and the use of assisted reproductive technologies. Second, although the diagnosis of OSA is made by polysomnography, we could not obtain the severity of OSA due to the de-identification in the database. Therefore, studies regarding the severity of OSA and subsequent female infertility are warranted. Third, although the study draws subjects from a large database, however, the number of infertility and OSA was only 33 and the number of OSA with no infertility in the control group was only 30 subjects. Fourth, there has no incidence of endometriosis or PCOS among infertile women in case group. A study has showed no infertility case in polycystic ovaries after performing blood hormones test and gynecologic ultrasonography and no constructive study that shows the relationship between OSA and endometriosis^44^. **Fifth, the duration of OSA is approximated by date of diagnosis code but we do not know whether or not it is treated subsequent to that.** Despite the listed limitations, our study provided a large group of patients and its longitudinal effects of over 14 years.

Reference:

44. Tong, C., Wang, Y. H., Yu, H. C., & Chang, Y. C. (2019). Increased Risk of Polycystic Ovary Syndrome in Taiwanese Women with Chronic Periodontitis: A Nationwide Population-Based Retrospective Cohort Study. Journal of women's health (2002), 28(10), 1436–1441. https://doi.org/10.1089/jwh.2018.7648

2. Secondly, you mention the low rate of PCOS (I think there was 1 case in the fertile group) and endometriosis but do not then provide any explanation and as above, I am concerned given another recent publication that you found so few cases of PCOS overall.

**Response:** Thank you for your thorough review and salient observations. We have advised it according to your recommendation.

This study had some limitations. First, our database could not provide detailed information about laboratory data or about the psychological impact of infertility and the use of assisted reproductive technologies. Second, although the diagnosis of OSA is made by polysomnography, we could not obtain the severity of OSA due to the de-identification in the database. Therefore, studies regarding the severity of OSA and subsequent female infertility are warranted. Third, although the study draws subjects from a large database, however, the number of infertility and OSA was only 33 and the number of OSA with no infertility in the control group was only 30 subjects. Fourth, there has no incidence of endometriosis or PCOS among infertile women in case group. **A study has showed no infertility case in polycystic ovaries after performing blood hormones test and gynecologic ultrasonography and no constructive study that shows the relationship between OSA and endometriosis^44^**. Fifth, the duration of OSA is approximated by date of diagnosis code but we do not know whether or not it is treated subsequent to that. Despite the listed limitations, our study provided a large group of patients and its longitudinal effects of over 14 years.

Reference:

44. Tong, C., Wang, Y. H., Yu, H. C., & Chang, Y. C. (2019). Increased Risk of Polycystic Ovary Syndrome in Taiwanese Women with Chronic Periodontitis: A Nationwide Population-Based Retrospective Cohort Study. Journal of women's health (2002), 28(10), 1436–1441. https://doi.org/10.1089/jwh.2018.7648

CONCLUSION

You conclude that OSA is more common in infertile women but left out what you had prior that women with OSA have a greater likelihood of being infertile so please add this back. I believe you have greater difficulty with the length of exposure given that you do not know if women are treated or not and should narrow your conclusion to the increased prevalence and likelihood and conclude that infertile women should be screened for signs and symptoms of OSA.

**Response:** Thank you for your thorough review and salient observations.

In conclusion, this study showed that OSA is more commonly seen in infertile women and increases the odds that a woman will be infertile. Therefore, infertile women should be screened for signs and symptoms of OSA, which may help to increase female fertility rate.

TABLES

Table 2: You do not mention what variables you adjusted for in the adjusted odds ratio. Please add that to the footnote at the bottom of the table. Delete the “without” and “with” headings under the associated disease as they are not necessary and it is confusing.

**Response:** Thank you for your thorough review and salient observations. We have revised them in Material and methods and Table section according to your recommendation.

We compared the study and control groups with regard to characteristics and common comorbidities, including hypertension, diabetes mellitus, hyperlipidemia, and COPD, by using chi-squared tests. The mean ages of the two groups were compared using Student’s t-test. The odds ratio (OR) for factors potentially associated with female infertility were evaluated using multivariable logistic regression with and without stratification. **The variables that adjusted in the odds ratio were the variables that listed in the table.** Correlation analysis was used to study the strength of a relationship between age groups and the variables listed in Table S2. All comparisons were two-tailed, and p-values <0.05 were considered statistically significant. The statistical analyses were performed using IBM SPSS v 22.0 software.

| **Table 2. Factors of infertility by using multivariable conditional logistic regression** | | | | | | | | |
| --- | --- | --- | --- | --- | --- | --- | --- | --- |
| **Variables** | **Crude OR** | **95% CI** | **95% CI** | ***P*** | **Adjusted OR** | **95% CI** | **95% CI** | ***P*** |
| **OSAs** | Reference |  |  |  | Reference |  |  |  |
|  | 1.900 | 1.056 | 3.417 | 0.001 | 2.101 | 1.118 | 3.950 | <0.001 |
| **Age group** |  |  |  |  |  |  |  |  |
| 20-25 | 1.000 | 0.818 | 1.193 | 0.999 | 0.947 | 0.772 | 1.160 | 0.753 |
| 26-30 | 1.000 | 0.821 | 1.188 | 0.999 | 0.852 | 0.697 | 1.040 | 0.179 |
| 31-35 | 1.000 | 0.804 | 1.213 | 0.999 | 0.781 | 0.626 | 0.989 | 0.050 |
| 36-40 | 1.000 | 0.739 | 1.320 | 0.999 | 0.783 | 0.572 | 1.071 | 0.165 |
| 41-45 | Reference |  |  |  | Reference |  |  |  |
| **Insured premium** |  |  |  |  |  |  |  |  |
| <18,000 | Reference |  |  |  | Reference |  |  |  |
| 18,000-34,999 | 0.858 | 0.544 | 1.352 | 0.536 | 1.047 | 0.640 | 1.712 | 0.759 |
| ≧35,000 | 0.861 | 0.459 | 1.618 | 0.663 | 0.782 | 0.396 | 1.543 | 0.511 |
| **HTN** | Reference |  |  |  | Reference |  |  |  |
|  | 1.860 | 0.537 | 2.379 | 0.532 | 1.745 | 0.450 | 2.264 | 0.315 |
| **DM** | Reference |  |  |  | Reference |  |  |  |
|  | 1.932 | 0.593 | 2.465 | 0.761 | 1.874 | 0.534 | 2.460 | 0.668 |
| **Hyperlipidemia** | Reference |  |  |  | Reference |  |  |  |
|  | 1.988 | 0.478 | 3.041 | 0.987 | 2.030 | 0.466 | 3.319 | 0.850 |
| **COPD** | Reference |  |  |  | Reference |  |  |  |
|  | 1.598 | 0.328 | 2.091 | 0.101 | 1.593 | 0.320 | 2.133 | 0.139 |
| **CKD** | Reference |  |  |  | Reference |  |  |  |
|  | 1.282 | 0.035 | 3.292 | 0.238 | 1.193 | 0.024 | 2.743 | 0.151 |
| **IHD** | Reference |  |  |  | Reference |  |  |  |
|  | 1.517 | 0.257 | 2.041 | 0.069 | 1.393 | 1.184 | 1.826 | 0.017 |
| **CHD** | Reference |  |  |  | Reference |  |  |  |
|  | 1.538 | 0.150 | 2.931 | 0.347 | 1.397 | 0.107 | 2.558 | 0.201 |
| **Stroke** | Reference |  |  |  | Reference |  |  |  |
|  | 1.564 | 0.117 | 3.717 | 0.479 | 1.449 | 0.087 | 3.442 | 0.371 |
| **Cancer** | Reference |  |  |  | Reference |  |  |  |
|  | 1.231 | 1.120 | 1.447 | <0.001 | 1.125 | 1.058 | 1.256 | <0.001 |
| **Obesity** | Reference |  |  |  | Reference |  |  |  |
|  | 1.705 | 0.253 | 2.959 | 0.511 | 1.744 | 0.258 | 3.206 | 0.627 |
| **Hyperestrogenism** | Reference |  |  |  | Reference |  |  |  |
|  | - | - | - | - | - | - | - | - |
| **Polycystic ovaries** | Reference |  |  |  | Reference |  |  |  |
|  | - | - | - | - | - | - | - | - |
| **Irregular menstrual cycle** | Reference |  |  |  | Reference |  |  |  |
|  | - | - | - | - | - | - | - | - |
| **Endometriosis** | Reference |  |  |  | Reference |  |  |  |
|  | - | - | - | - | - | - | - | - |
| **Uterine leiomyoma** | Reference |  |  |  | Reference |  |  |  |
|  | - | - | - | - | - | - | - | - |
| **Cushing's syndrome** | Reference |  |  |  | Reference |  |  |  |
|  | - | - | - | - | - | - | - | - |
| **Thyrotoxicosis with or without goiter** | Reference |  |  |  | Reference |  |  |  |
|  | 2.414 | 0.727 | 3.749 | 0.286 | 1.877 | 0.437 | 2.796 | 0.778 |
| **Acquired hypothyroidism** | Reference |  |  |  | Reference |  |  |  |
|  | 2.098 | 0.367 | 4.280 | 0.840 | 1.917 | 0.281 | 4.061 | 0.914 |
| **Anxiety** | Reference |  |  |  | Reference |  |  |  |
|  | 1.759 | 0.270 | 3.132 | 0.610 | 1.879 | 0.272 | 3.904 | 0.859 |
| **Depression** | Reference |  |  |  | Reference |  |  |  |
|  | 1.600 | 0.257 | 2.400 | 0.246 | 1.644 | 0.253 | 2.689 | 0.404 |
| **Tobacco use disorder** | Reference |  |  |  | Reference |  |  |  |
|  | 1.852 | 0.896 | 2.571 | 0.184 | 1.829 | 0.885 | 2.540 | 0.182 |
| **Alcoholism** | Reference |  |  |  | Reference |  |  |  |
|  | 1.532 | 0.722 | 2.386 | 0.223 | 1.513 | 0.714 | 2.357 | 0.220 |
| **Season** |  |  |  |  |  |  |  |  |
| Spring | Reference |  |  |  | Reference |  |  |  |
| Summer | 1.018 | 0.875 | 1.186 | 0.684 | 0.980 | 0.831 | 1.154 | 0.937 |
| Autumn | 0.960 | 0.824 | 1.118 | 0.702 | 0.938 | 0.796 | 1.105 | 0.622 |
| Winter | 0.871 | 0.746 | 1.017 | 0.110 | 0.823 | 0.696 | 0.972 | 0.044 |
| **Location** |  |  |  |  |  | | | |
| Northern Taiwan | Reference |  |  |  | **Had multicollinearity with urbanization level** | | | |
| Middle Taiwan | 0.794 | 0.696 | 0.906 | 0.001 | **Had multicollinearity with urbanization level** | | | |
| Southern Taiwan | 0.865 | 0.751 | 0.996 | 0.045 | **Had multicollinearity with urbanization level** | | | |
| Eastern Taiwan | 0.871 | 0.659 | 1.153 | 0.377 | **Had multicollinearity with urbanization level** | | | |
| Outlets islands | 0.899 | 0.419 | 1.929 | 0.799 | **Had multicollinearity with urbanization level** | | | |
| **Urbanization level** |  |  |  |  |  |  |  |  |
| 1 (The highest) | 3.459 | 2.808 | 4.262 | <0.001 | 1.639 | 1.304 | 2.060 | <0.001 |
| 2 | 2.003 | 1.624 | 2.469 | <0.001 | 1.182 | 0.947 | 1.475 | 0.087 |
| 3 | 0.900 | 0.665 | 1.218 | 0.539 | 0.907 | 0.667 | 1.235 | 0.629 |
| 4 (The lowest) | Reference |  |  |  | Reference |  |  |  |
| **Level of care** |  |  |  |  |  |  |  |  |
| Hospital center | 6.324 | 5.418 | 7.381 | <0.001 | 5.734 | 4.841 | 6.791 | <0.001 |
| Regional hospital | 2.272 | 1.939 | 2.662 | <0.001 | 2.322 | 1.974 | 2.730 | <0.001 |
| Local hospital | Reference |  |  |  | Reference |  |  |  |
| **OR= odds ratio, CI = confidence interval, Adjusted OR: Adjusted variables listed in the table** | | | | | | | | |

Tables 3 and 4: I do not understand what is meant by first and last exposure to OSA prior to the diagnosis of infertility. Given how speculative this information is, you might to consider deleting it since you do not know if the OSA is treated or not.

**Response:** Thank you for your thorough review and salient observations. We have deleted Table 3 and 4 according to your recommendation.

MINOR WEAKNESSES

INTRODUCTION

Second paragraph: 1. website reference to definition of infertility should be a footnote; 2. The reduction in fertility rate could be a function of societal change and not necessarily indicate a change in rate of infertility. Consider rephrasing or eliminating that discussion.

**Response:** Thank you for your thorough review and salient observations. We have revised them according to your recommendation.

According to the American College of Obstetricians and Gynecologists (ACOG), the definition of infertility is the failure to conceive after 1 year or more of regular unprotected sexual intercourse^6^. The prevalence of infertility has increased since 1990; in 2010, approximately 48.5 million individuals worldwide suffered from infertility^7^. Moreover, according to the data published by Taiwan’s Ministry of the Interior, the fertility rates of women within childbearing age decreased from 1.680 births per woman in 2000 to 1.080 births per woman in 2018, with the mean age of the women at their first birth increasing from 22.88 years to 30.90 years during that period. Female infertility can result from various conditions, including endometriosis, pelvic adhesion, polycystic ovary syndrome, tubal blockage, hyperprolactinemia, and congenital or acquired uterine or ovarian abnormalities^8^.

References:

6. Medicine CoGPftASfR, Obstetricians tACo, Gynecologists. Infertility workup for the Women’s Health Specialist. Obstet Gynecol 2019; 133: e377-e84.

7. Mascarenhas MN, Flaxman SR, Boerma T, Vanderpoel S, Stevens GA. National, regional, and global trends in infertility prevalence since 1990: a systematic analysis of 277 health surveys. PLoS Med 2012; 9(12): e1001356.

8. Abrao MS, Muzii L, Marana R. Anatomical causes of female infertility and their management. International journal of gynaecology and obstetrics: the official organ of the International Federation of Gynaecology and Obstetrics 2013; 123 Suppl 2: S18-S24.

Third paragraph: “Infertility can be associated with multiple factors”.

**Response:** Thank you for your thorough review and salient observations. We have revised it according to your recommendation.

MATERIALS AND METHODS

Study Population, first paragraph: The diagnosis of OSA is made BY polysomnography.

**Response:** Thank you for your thorough review and salient observations. We have revised it according to your recommendation.

RESULTS

First Paragraph: Move the sentence about the prevalence of women of different age groups before the sentence about the prevalence of OSA as it interrupts the flow.

**Response:** Thank you for your thorough review and salient observations. We have revised it according to your recommendation.

We identified 4,078 female patients in the database who had been diagnosed with infertility by the end of the study. After applying the exclusion criteria, 2,400 patients were included and assigned as the study group in the analysis. The control group comprised 4,800 matched women without infertility. The mean ages in the study groups were 32.19 ± 6.20 years, whereas the mean ages in the control groups were 32.24 ± 6.37 years. Table 1 summarizes the characteristics of the study and the control groups. **There were significantly more patients who had been diagnosed with OSA in the study group than in the control group (1.38% vs. 0.63%; p = .002). In addition, we have found that 33 patients in the study group and 30 patients in the reference group, who have had prior exposure to OSA. Women aged 26-30 years and 31-35 years were more than other aged groups in our study population (28.0% and 32.29% respectively).**

DISCUSSION:

6th paragraph: Please edit the sentence about the cows to be grammatically correct.

**Response:** Thank you for your thorough review and salient observations. We have deleted the paragraph because the discussion about seasonality is less relevant.

Last, we are deeply honored by the time and effort you spent in reviewing this manuscript. In reviewing and revising our text, we are motivated to read more and thus learn more from your criticisms.
