# Supplementary material for: Obstructive sleep apnea increases risk of female infertility: A 14-year nationwide population-based study
Source: PLoS One. 2021 Dec 15;16(12):e0260842. doi: 10.1371/journal.pone.0260842 (PMC8673645; doi:10.1371/journal.pone.0260842)
Supplement: Attachment — Submitted filename: Response to Reviewers 2021-11-03.docx [file pone.0260842.s005.docx]

**Answer to Editor’s and Reviewer's comments**

Thank you for your positive comments on this manuscript. The responses to the raised questions are below.

Reviewer's Responses to Questions

**Comments to the Author**

1. If the authors have adequately addressed your comments raised in a previous round of review and you feel that this manuscript is now acceptable for publication, you may indicate that here to bypass the “Comments to the Author” section, enter your conflict of interest statement in the “Confidential to Editor” section, and submit your "Accept" recommendation.

Reviewer #2: (No Response)

2. Is the manuscript technically sound, and do the data support the conclusions?

The manuscript must describe a technically sound piece of scientific research with data that supports the conclusions. Experiments must have been conducted rigorously, with appropriate controls, replication, and sample sizes. The conclusions must be drawn appropriately based on the data presented.

Reviewer #2: Yes

**Response:** Thank you for your thorough review and salient observations.

3. Has the statistical analysis been performed appropriately and rigorously?

Reviewer #2: Yes

**Response:** Thank you for your thorough review and salient observations.

4. Have the authors made all data underlying the findings in their manuscript fully available?

The PLOS Data policy requires authors to make all data underlying the findings described in their manuscript fully available without restriction, with rare exception (please refer to the Data Availability Statement in the manuscript PDF file). The data should be provided as part of the manuscript or its supporting information, or deposited to a public repository. For example, in addition to summary statistics, the data points behind means, medians and variance measures should be available. If there are restrictions on publicly sharing data—e.g. participant privacy or use of data from a third party—those must be specified.

Reviewer #2: Yes

**Response:** Thank you for your thorough review and salient observations.

5. Is the manuscript presented in an intelligible fashion and written in standard English?

PLOS ONE does not copyedit accepted manuscripts, so the language in submitted articles must be clear, correct, and unambiguous. Any typographical or grammatical errors should be corrected at revision, so please note any specific errors here.

Reviewer #2: Yes

**Response:** Thank you for your thorough review and salient observations.

6. Review Comments to the Author

Please use the space provided to explain your answers to the questions above. You may also include additional comments for the author, including concerns about dual publication, research ethics, or publication ethics. (Please upload your review as an attachment if it exceeds 20,000 characters)

Reviewer #2: Thank you for your attention to my previous comments. This paper is much improved. There are still some minor weaknesses to address but we are almost there. Please see remaining comments below.

**Response:** Thank you for your thorough review and salient observations. We have revised these mistakes according to your suggestion.

**Minor Weaknesses**

Abstract: delete the sentence in the conclusion about the likelihood of being infertile increasing with time from diagnosis of OSA as you deleted this analysis because of insufficient information.

**Response:** Thank you for your thorough review and salient observations. We have revised these mistakes according to your suggestion.

Conclusion: Our study showed OSA is more commonly seen in infertile women, especially those with concomitant comorbidities, but yet its risk is often underdiagnosed. Therefore, early detection of OSA in women, especially in those with atypical symptoms, may help to increase female fertility rate.

Introduction, 3rd paragraph: please delete the sentence about CPAP improving erectile function—not relevant to a population of women (and be careful about adjusting your references).

**Response:** Thank you for your thorough review and salient observations. We have revised these mistakes according to your suggestion.

Infertility was associated with multiple factors, such as inflammation, obesity, intermittent hypoxia and sympathetic activation. Scientists have demonstrated young to middle-aged mice which experienced chronic intermittent hypoxia, have reduced the reproductive functions via the decreased of nitric oxide production. They have found the association of male infertility in these OSA mice^8,9^. In our previous study, we have found an increased risk of infertility in female patients with non-apnea sleep disorder^10^. However, little is known about whether OSA is associated with a risk of female infertility.

References:

8.	Torres M, Laguna-Barraza R, Dalmases M, et al. Male fertility is reduced by chronic intermittent hypoxia mimicking sleep apnea in mice. Sleep 2014; 37(11): 1757-65.

9.	Soukhova-O'Hare GK, Shah ZA, Lei Z, Nozdrachev AD, Rao CV, Gozal D. Erectile dysfunction in a murine model of sleep apnea. American journal of respiratory and critical care medicine 2008; 178(6): 644-50.

10.	Wang ID, Liu YL, Peng CK, et al. Non-apnea sleep disorder increases the risk of subsequent female infertility-a nationwide population-based cohort study. Sleep 2018; 41(1): zsx186.

Discussion, 1st paragraph, second sentence: “ . . . found that infertile women had” should be “infertile women have” and 3rd sentence: . . . which our groups have shown” or “which our group has shown”—whichever version is correct.

**Response:** Thank you for your thorough review and salient observations. We have revised these mistakes according to your suggestion.

Our study is the largest retrospective study to date demonstrating the association of female infertility with OSA. In this nationwide, population-based, case-control study of over 14 years, we have found that infertile women had an odds ratio of 2.1 of having OSA compared with women who were fertile. This article is the second study in which our groups has shown that women with sleep disorders could link to female infertility. In our previous article, we have found that women with non-apnea sleep disorder have a 3.718-fold risk of female infertility compared with the control cohort^10^.

Reference:

10.	Wang ID, Liu YL, Peng CK, et al. Non-apnea sleep disorder increases the risk of subsequent female infertility-a nationwide population-based cohort study. Sleep 2018; 41(1): zsx186.

Discussion, 3rd paragraph: As mentioned before, the first sentence is incorrect. Women with PCOS are at increased risk for OSA; women with OSA are NOT known to be at increased risk for PCOS. Your topic sentence really should be about a theoretical relationship of hormonal dysregulation from OSA leading to infertility. Please change it.

**Response:** Thank you for your thorough review and salient observations. We have revised these mistakes according to your suggestion.

Patients with OSA are at risk of metabolic disorders, including an irregular menstrual cycle, endometriosis, and uterine leiomyoma^27^.

Reference:

27.	Skatrud J, Dempsey J, Kaiser D. Ventilatory response to medroxyprogesterone acetate in normal subjects: time course and mechanism. J Appl Physiol 1978; 44(6): 939-44.

Discussion, 3rd paragraph: . . . “ . . . cortisol31. The increase in cortisol levels can downregulate the HPA axis and inhibit GnRH at the pituitary level, which may alter sex hormone profiles and thus lead to infertility.32”

**Response:** Thank you for your thorough review and salient observations. We have revised these mistakes according to your suggestion.

Discussion, 3rd paragraph: delete the discussion about salivary cortisol. It does not add anything and is confusing.

**Response:** Thank you for your thorough review and salient observations. We have deleted the discussion about salivary cortisol according to your suggestion.

Discussion 4th paragraph: “ . . . prevalence of OSA In women is lower than IN men”.

**Response:** Thank you for your thorough review and salient observations. We have revised these mistakes according to your suggestion.

Discussion 4th paragraph: the lower prevalence of OSA in pre-menopausal women is not a function of atypical presentation, it simply is. The atypical presentation explains the underdiagnosis of women but the prevalence is still lower in women so please revise this section.

**Response:** Thank you for your thorough review and salient observations. We have revised these mistakes according to your suggestion.

It is because of atypical OSA symptoms such as depression, headache, anxiety and insomnia are more frequent presented in women^31^.

Reference:

31. Collop NA, Adkins D, Phillips BA. Gender differences in sleep and sleep-disordered breathing. Clin Chest Med 2004; 25(2): 257-68.

Limitations: Delete the first limitation.

**Response:** Thank you for your thorough review and salient observations. We have revised these mistakes according to your suggestion.

Limitations: fourth limitation: please point out that the absence of women with endometriosis and PCOS in the infertile cohort potentially limits the applicability in populations where these disorders are more common. Please delete the sentence about no infertility in PCOS and reference 45—not the point here.

**Response:** Thank you for your thorough review and salient observations. We have revised these mistakes according to your suggestion.

Limitations: please delete the fifth limitation as you have removed that analysis from this paper.

**Response:** Thank you for your thorough review and salient observations. We have revised these mistakes according to your suggestion.

7. PLOS authors have the option to publish the peer review history of their article (what does this mean?). If published, this will include your full peer review and any attached files.

If you choose “no”, your identity will remain anonymous but your review may still be made public.

Do you want your identity to be public for this peer review? For information about this choice, including consent withdrawal, please see our Privacy Policy.

Reviewer #2: No

**Response:** Thank you for your thorough review and salient observations.

Last, we are deeply honored by the time and effort you spent in reviewing this manuscript. In reviewing and revising our text, we are motivated to read more and thus learn more from your criticisms.
