# Supplementary material for: Obstructive sleep apnea increases risk of female infertility: A 14-year nationwide population-based study
Source: PLoS One. 2021 Dec 15;16(12):e0260842. doi: 10.1371/journal.pone.0260842 (PMC8673645; doi:10.1371/journal.pone.0260842)
Supplement: Attachment — Submitted filename: 2021-11-17 Response to Reviewers.docx [file pone.0260842.s006.docx]

**Answer to Editor’s and Reviewer's comments**

Thank you for your positive comments on this manuscript. The responses to the raised questions are below.

Reviewer's Responses to Questions

**Comments to the Author**

The academic editor went through the latest revisions for the reviewer and most of have been changed in a satisfactory manner. However, there is still one small change to make, regarding the comment on prevalence (5th paragraph of discussion). The reviewer was pointing out that the lower prevalence in women is NOT due to an atypical presentation as epidemiologic studies, in which all subjects are studied, is lower in women. However, what is lower is underevaluation because of atypical presentations. Thus, the second sentence of this paragraph should be changed to: 'There is also likely underdiagnosis of OSA in women due to atypical symptoms such as...'.

**Response:** Thank you for your thorough review and salient observations. We have revised these mistakes according to your suggestion.

Scientists have demonstrated the prevalence of OSA in women is lower than in men^2^. There is also likely underdiagnosis of OSA in women due to atypical symptoms such as depression, headache, anxiety and insomnia are more frequent presented in women^31^.

References:

2. Young T, Palta M, Dempsey J, Skatrud J, Weber S, Badr S. The occurrence of sleep-disordered breathing among middle-aged adults. The New England journal of medicine 1993; 328(17): 1230-5.

31. Ferin M. Stress and the Reproductive Cycle. The Journal of Clinical Endocrinology & Metabolism 1999; 84(6): 1768-74.

Last, we are deeply honored by the time and effort you spent in reviewing this manuscript. In reviewing and revising our text, we are motivated to read more and thus learn more from your criticisms.
